# Regulatory role and therapeutic prospect of lactate modification in cancer

**DOI:** 10.3389/fphar.2025.1508552

**Published:** 2025-02-17

**Authors:** Mengdi Sun, Kejing Wang, Fang Lu, Donghua Yu, Shumin Liu

**Affiliations:** ^1^ Graduate School, Heilongjiang University of Chinese Medicine, Harbin, China; ^2^ Institute of Traditional Chinese Medicine, Heilongjiang University of Chinese Medicine, Harbin, China

**Keywords:** lactate, lactylation modification, cancer, tumor microenvironment, immune cells, cancer treatment

## Abstract

Post-translational modifications (PTMs) of proteins refer to the process of adding chemical groups, sugars, or other molecules to specific residues of target proteins following their biosynthesis by ribosomes. PTMs play a crucial role in processes such as signal transduction, epigenetics, and disease development. Lactylation is a newly discovered PTM that, due to its close association with lactate—the end product of glycolytic metabolism—provides a new perspective on the connection between cellular metabolic reprogramming and epigenetic regulation. Studies have demonstrated that lactylation plays a significant role in tumor progression and is associated with poor clinical prognosis. Abnormal histone lactylation can influence gene expression in both tumor cells and immune cells, thereby regulating tumor progression and immunosuppression. Lactylation of non-histone proteins can also modulate processes such as tumor proliferation and drug resistance. This review summarizes the latest research progress in the field of lactylation, highlighting its roles and mechanisms in tumorigenesis, tumor development, the tumor microenvironment, and immunosuppression. It also explores the potential application value of lactylation in tumor-targeted therapy and combined immunotherapy.

## 1 Introduction

PTMs of proteins involve covalent modifications on the protein backbone or amino acid side chains, thereby increasing complexity from the genomic to the proteomic level, are pivotal in generating proteomic diversity. Common forms include phosphorylation, glycosylation, methylation, ubiquitination, etc (Wang H. et al., 2023). Notably, histone PTMs can modulate nucleosome interactions or recruit non-histone proteins, representing a critical class of epigenetic modifications (Millán-Zambrano et al., 2022). Research has demonstrated that PTMs play a vital role in regulating tumor immune escape and influencing the efficacy of immunotherapy (Pan and Chen, 2022).

The modification substrates of PTMs predominantly originate from intracellular metabolic pathways (Soriano-Baguet and Brenner, 2023). For instance, acetyl coenzyme A, a key metabolite generated through processes such as the oxidative decarboxylation of pyruvate and β-oxidation of fatty acids, serves as the substrate for histone acetylation (Sabari et al., 2017); S-adenosylmethionine, synthesized in cells via the action of methionine adenosyltransferase on adenosine triphosphate (ATP) and methionine, functions as a methyl donor to regulate protein methylation (Guccione and Richard, 2019). Additionally, succinyl coenzyme A, produced by the tricarboxylic acid cycle, provides the substrate for protein succinylation. Furthermore, S-nitrosothiols, formed by the reaction of nitric oxide with free cysteine residues, serve as substrates for S-nitrosylation. Intracellular lactate primarily results from glycolysis. Once considered merely a metabolic waste product, it is now recognized as an essential regulator of multiple signaling pathways in both normal tissues and tumors (Haas et al., 2016). The abnormal proliferation and metabolic reprogramming of tumor cells lead to hypoxia and nutrient deprivation in the tumor microenvironment (TME) (Jan et al., 2024), resulting in elevated lactate levels within tumor cells and the TME (Gao et al., 2022). Recent studies have demonstrated that lactate shuttles between various cell types in the TME, including tumor cells, cancer-associated fibroblasts (CAFs), tumor-associated macrophages (TAMs), and tumor-infiltrating lymphocytes (TILs) (Wang et al., 2020), thereby facilitating the establishment of a microenvironment conducive to tumor growth and immune evasion (Dai et al., 2024). Currently, the primary treatment modalities for cancer include surgery, chemotherapy, and radiotherapy. However, anti-cancer drugs used in clinical practice commonly exhibit issues such as adverse reactions, limited selectivity, and drug resistance (Batool et al., 2024). This review summarizes recent advances in lactylation modification, encompassing both histone and non-histone lactylation in tumor development and intercellular interactions within the microenvironment. It also explores the potential of targeting lactate metabolism and lactylation-related enzymes as therapeutic strategies for cancer treatment, offering insights into developing novel combined targeted therapy and immunotherapy regimens.

## 2 Lactylation modification and associated proteins

### 2.1 Discovery of lactylation modification

In the early 1920s, Warburg and his research team (Warburg, 1956; Koppenol et al., 2011) revealed a thought-provoking phenomenon: even under non-hypoxic conditions, cancer cells can produce lactate from glucose to maintain the basic energy required for life. Concurrently, due to mitochondrial dysfunction and limited production of ATP under hypoxic conditions, the Warburg effect increases the lactate production rate and also causes acidification of the tumor microenvironment, thus creating favorable conditions for the rapid proliferation and growth of tumor cells (Xin et al., 2022). This discovery has overturned the previous traditional understanding of tumor cell metabolism. Lactate serves as both a secondary product of energy metabolism and a crucial molecule in signaling.

Histones are crucial components of chromosomes, comprising core histones (H2A, H2B, H3, and H4) and linker histones (H1 and H5). These proteins possess a positive charge that facilitates their interaction with negatively charged DNA to form nucleosomes. Histone PTMs play a pivotal role in epigenetic regulation. Through covalent modifications, various acyl groups can be attached to the amino acid residues of histones, thereby altering the molecular structure of nucleosomes and influencing the recruitment of effector proteins or directly modulating the transcription of target genes (Millán-Zambrano et al., 2022). Histone lactylation modification is a new type of histone PTM reported in 2019. Zhang et al. (2019) demonstrated that under conditions of elevated endogenous lactate levels due to hypoxia or bacterial infection, the degree of histone lactylation in cells significantly increased. Conversely, inhibiting the enzymatic activity of key glycolytic enzymes or lactate dehydrogenase (LDH) resulted in a decrease in histone lactylation, indicating a direct correlation between histone lactylation and both glycolysis and intracellular lactate concentrations. Functional studies have shown that during M1 macrophage polarization, histone lactylation directly activates the expression of wound-healing-related genes through a p53-dependent and histone acetyltransferase p300-mediated mechanism in response to hypoxic or inflammatory stimuli. To date, numerous histone lactylation sites have been identified, primarily on lysine residues. Notably, H3K18la, H4K5la, and H4K18la have been extensively studied (Qu et al., 2023) (Table 1). Furthermore, lactylation can also occur in non-histone proteins, influencing their structure and function and thereby playing diverse roles (Zhang et al., 2021; Mao et al., 2024). For instance, Yang et al. (2023b) identified 9275 lactylation sites in liver cancer cells, with 9256 located on non-histone proteins. Further studies revealed that lactylation at position K28 of adenylate kinase 2 (AK2) promotes tumor cell proliferation and metastasis by modulating the activity of the p53 pathway.

**TABLE 1:** Mechanism of lactate modification in cancer.

Disease type	Modification site/enzyme	Target gene	Action mechanism	Reference
Ocular melanoma	H3K18/p300	YTHDF2	YTHDF2 is upregulated by H3K18 lactoacylation and promotes tumorigenesis by inhibiting TP53 and PER1	Yu et al. (2021)
Non-small cell lung cancer	H4K8	HK-1/IDH3G	Glycolytic enzymes (HK-1, PKM) and TCA circulating enzymes (SDHA, IDH3G) are downregulated and upregulated by lactic acid, respectively, and histone lactation is increased in the HK-1 and IDH3G promoters	Jiang et al. (2021)
Colon cancer	H3K18	METTL3	Lactate accumulation in the tumor microenvironment induces upregulation of METTL3 in tumor infiltrating myeloid cells (TIMs) through H3K18 lactation	Xiong et al. (2022)
Hepatocellular carcinoma (HCC)	K348/SIRT3	CCNE2	SIRT3 deemulsifies CCNE2 K348la and promotes apoptosis of HCC cells, preventing the growth of HCC *in vivo*	Jin et al. (2023)
Hepatocellular carcinoma (HCC)	K28 (AK2)/K413(IDH2)	AK2/IDH2	The acylation of K28 inhibits the function of AK2 and promotes the proliferation and metastasis of HCC cells	Yang et al. (2023b)
Gastric/lung cancer	H3K18la	NFAT1	Treg cells actively absorb lactic acid through monocarboxylic transporter 1 to promote the transfer of NFAT1 into the nucleus, thereby enhancing the expression of PD-1 and activating immune checkpoints	Kumagai et al. (2022)
Colorectal cancer	H3K18la	RUBCNL	Tumor-derived lactic acid promotes the expression of the autophagy enhancing protein RUBCNL through histone H3 lysine-18 lactation (H3K18la), thereby promoting the resistance of colorectal cancer to bevacizumab therapy	Li et al. (2024b)
Hepatocellular carcinoma	Lys72	MOESIN	Lactate regulates the production of Treg cells through the emulsification of Lys72 in MOESIN, thereby improving the interaction of MOESIN with TGF-β receptor I and downstream SMAD3 signaling pathways	Gu et al. (2022)
Gastric cancer	2375 Kla sites in 1014 proteins	-	Lactic acid affects RNA splicing in AGS cells of gastric cancer	Yang et al. (2022a)
Cervical cancer	H3K9/18/23/27/79	-	-	Zhang et al. (2019)
Breast cancer	H4K12lac	Ki-67	It is mainly related to DNA replication and deoxyribonuclease activity regulation, and the main pathways involved are synaptic vesicle cycle, base excision repair and iron death	Cui et al. (2024)
Breast cancer	-	RAD51/NEK10/PCP2/IDO1/CASP14/CLSTN2/IGHG1	Involved in homologous DNA recombination and repair; Regulates the cell cycle, promotes MEK1 activation, leads to G2/M phase block and ERK1/2 phosphorylation; Immunosuppressive TME; Participate in apoptosis	Jiao et al. (2024)
Carcinoma of bladder	H3K18la	YBX1/YY1	H3K18la is upregulated in cisplatin-resistant bladder cancer cell lines and increases the expression of transcription factors such as YBX1 and YY1, ultimately driving cisplatin resistance in BCa	Li et al. (2024a)
Prostate cancer	-	KIAA1199/HIF1α/MCT1	KIAA1199 promotes angiogenesis by increasing hyaluronic acid; HIF1α increased the transcriptional activity of KIAA1199 under normal oxygen condition. McT1-mediated lactate enhanced HIF1α lactoacylation to stimulate KIAA1199 signaling	Luo et al. (2022)

### 2.2 Proteins associated with lactylation modification

In recent years, studies on the mechanism of lactylation modification have revealed that it is a dynamic and reversible process, jointly regulated by specific lactyl transferases “writers” and delactylases “erasers,” which add or remove lactyl groups from modified lysine residues. Effector proteins known as “readers” specifically recognize and bind to these modifications, influencing downstream signaling pathways and regulating various biological processes (Wang J.-H. et al., 2023). P300, a classic histone acetyltransferase (HAT), catalyzes various types of protein modifications and plays a crucial role in the development of malignant tumors (Zeng et al., 2023). Zhang et al. (2019) demonstrated that p300 also functions as a lactylation writer, transferring L-lactate to histones; overexpression of p300 significantly increases intracellular lactylation levels. Histone deacetylases (HDACs), including two families—HDAC1-11 and silent information regulator 1–7 (SIRT1-7)—mediate lysine deacetylation. Moreno-Yruela et al. (2022) found that HDACs also act as histone lysine delactylases. *In vitro* studies have shown that HDAC1-3 can significantly reduce H3K18la and H4K5la levels, while SIRT1-3 slightly decrease these levels. Knockdown of HDAC1 and HDAC3 in cells leads to increased H4K5la levels, but total lactylation and H3K18la levels remain unchanged. Simultaneous knockdown of HDAC1-3 has a significant impact on H4K5la levels. The discrepancies between *in vitro* and intracellular experimental results suggest the presence of cofactors that confer site-specific delactylation activity to HDACs, although no relevant research has been reported thus far. In summary, as a novel type of PTM, our current understanding of lactylation modification sites, modification processes, and related proteins remains limited, necessitating further in-depth research.

### 2.3 The relationship between lactylation modification and other PTMs

The relationship between lactylation and other PTMs provides new insights into the interaction between metabolic and epigenetic processes. Both acetylation and lactylation are regulated by HATs and HDACs, and studies have demonstrated a close association between these two modifications. Yang K. et al. (2022) found that lactate can simultaneously increase the levels of both lactylation and acetylation of high mobility group protein B1 (HMGB1) in macrophages. Lactylation is also related to other histone modifications, such as crotonylation and butyrylation. Dai et al. (2022) reported that lactylation and crotonylation exhibit synergistic effects on neural differentiation and cell proliferation. Additionally, lactylation can be associated with butyric acid-mediated butyrylation. Xie et al. (2022) discovered that butyric acid promotes an increase in protein lactylation levels in HeLa cells, while inhibiting HDACs significantly diminishes this effect. However, current research on the relationship between lactylation and other PTMs is still in its early stages and requires further investigation.

## 3 The role of lactate in tumors

### 3.1 Lactate metabolism

Under normal conditions, intracellular glucose ultimately produces a large amount of ATP and CO_2_ through glycolysis and oxidative phosphorylation (OXPHOS) (Sokolov et al., 2015). Under stress conditions such as hypoxia, exercise, trauma, sepsis, and heart failure, cells undergo anaerobic glycolysis, where pyruvate produced by glycolysis is catalyzed by LDH in the cytoplasm to produce lactate and release a small amount of energy (Hermans et al., 2020). Warburg, (1956) observed that tumor cells also undergo anaerobic glycolysis and secrete large amounts of lactate even in the presence of sufficient oxygen, a phenomenon known as aerobic glycolysis or the “Warburg effect.” Additionally, tumor cells can produce lactate through glutamine catabolism (DeBerardinis et al., 2007). Although glycolysis and glutamine catabolism generate much less energy than OXPHOS, the various metabolic intermediates produced by these processes provide raw materials for the synthesis of macromolecules such as nucleotides, purines, pyrimidines, amino acids, and fatty acids, thereby meeting the needs of rapidly proliferating tumor cells (Pavlova et al., 2022).

**FIGURE 1:**
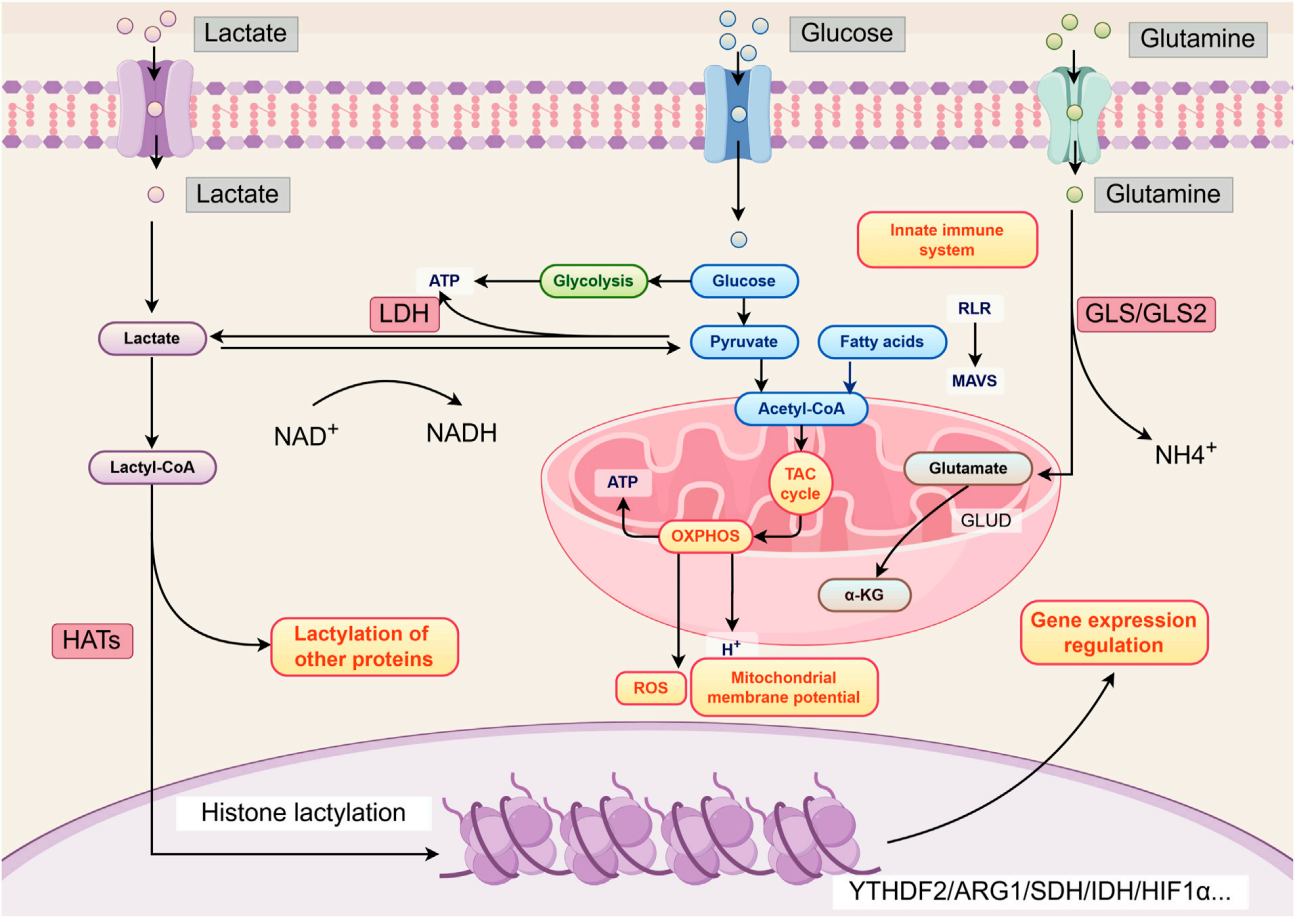
Lactate production and function. By Figdraw.

The “Warburg effect” in tumor cells results from genetic changes and abnormal expression of glycolysis-related enzymes and lactate transporters. Lactate transport across the cell membrane is mediated by specific receptors on the cell surface, primarily including monocarboxylate transporters (MCTs), G protein-coupled receptors (GPRs) such as GPR81, and hydroxycarboxylic acid receptor 1 (HCAR1) found in brain cells (Hadzic et al., 2020). The exchange of lactate between the intracellular and extracellular environments is mainly mediated by MCT1 and MCT4 (Payen et al., 2020). MCT1 predominantly mediates lactate uptake, while MCT4 facilitates the efflux of intracellular lactate. Studies have shown that inhibiting the expression of MCT1 and MCT4 can effectively suppress tumor growth by interfering with lactate metabolism (Doherty and Cleveland, 2013). GPR81, a lactate receptor on the cell membrane, can directly activate multiple intracellular signaling pathways in response to extracellular lactate stimulation (Brown and Ganapathy, 2020). Hypoxia-inducible factor-1α (HIF-1α) and c-Myc are two key transcription factors that maintain high-level glycolysis in tumor cells. They regulate lactate production through various mechanisms, such as upregulating the expression of hexokinase 2 (HK2), pyruvate kinase muscle isoform 2 (PKM2), and LDHs in glycolysis, and inhibiting mitochondrial pyruvate metabolism (Gordan et al., 2007). Additionally, they regulate the expression of MCT1 and MCT4 to promote lactate efflux into the TME, thereby preventing intracellular acidification (Doherty and Cleveland, 2013). Moreover, the “Warburg effect” in tumor cells is also influenced by the activity of mammalian target of rapamycin (mTOR). mTOR senses and integrates various environmental signals to control cell growth and proliferation, serving as a key regulatory node in the cellular metabolic network. Research has demonstrated that activation of the mTOR pathway increases the expression of HIF-1α and c-Myc, further upregulating glucose transporter 1 (GLUT1), glycolysis-related enzymes, LDHA, MCT1, and MCT4, thereby promoting lactate production in tumor cells (Mossmann et al., 2018) (Figure 1).

### 3.2 Elevated lactate levels and their roles in the tumor microenvironment

Tumor cells can excrete lactate produced by aerobic glycolysis into the extracellular space through MCTs to prevent intracellular acidification. Additionally, studies have shown that other cellular components in the TME, such as CAFs, also metabolize and secrete lactate (Lee and Yoon, 2015). This leads to increased lactate levels in the TME (Parks and Pouysségur, 2017). The lactate concentration in the serum of healthy individuals is typically 1.5–3 mmol/L, while in tumor patients, it can increase to 10–30 mmol/L, and even reach up to 50 mmol/L within tumors (Brizel et al., 2001). Studies have demonstrated that high lactate concentrations in the TME are associated with poor clinical outcomes, including lymph node or distant metastasis and low survival rates in various cancers (Dhup et al., 2012). Lactate in the TME can affect immune cell function, stimulate angiogenesis, and promote TME remodeling through multiple mechanisms, enabling tumors to evade immune surveillance (Pérez-Tomás and Pérez-Guillén, 2020). Moreover, lactate is closely related to the states and functions of other cellular components in the TME, including various immune cells, CAFs, and endothelial cells, further regulating processes such as immunosuppression, invasion, and metastasis (Wang Z.-H. et al., 2021; Gao et al., 2022).

#### 3.2.1 Effects of elevated lactate levels on the functions of immune cells

Numerous studies have confirmed that lactate not only provides energy for tumor metabolism but also exerts immunosuppressive effects in the local lactate environment of tumors, with a correlation between its concentration and these effects (Chen et al., 2022; Zhang et al., 2022). Tumor cells exhibit high glycolytic activity (Li et al., 2022), leading to continuous glucose consumption during growth. This depletes glucose levels in the TME, resulting in insufficient energy supply for T cells, reduced T cell activity, and decreased production of key cytokines such as interferon-γ (IFN-γ), thereby limiting T cell-mediated tumor killing. Moreover, lactate produced during tumor glycolysis accumulates in the TME, causing acidification. The acidic environment inhibits T cell function and promotes the formation of an immunosuppressive microenvironment. Accumulated lactate can also induce the differentiation and activation of myeloid-derived suppressor cells (MDSCs), regulatory T cells (Tregs), and TAMs. These cells secrete immunosuppressive factors, inhibit the immune responses of natural killer (NK) cells and T cells, help tumor cells evade immune surveillance, and promote tumor growth (Zhang et al., 2022).

Research shows that the acidic environment with a pH of 6.0–6.6, induced by high-concentration lactate, can promote tumor cell proliferation, invasion, metastasis, evasion of apoptosis, and immune escape (Audero et al., 2022). Carbonic anhydrase IX (CAIX) is a key enzyme enabling solid tumor cells to adapt and survive under hypoxic conditions. It functions as an extracellular pH regulator, maintaining an acidic extracellular pH through the catalysis of lactate production, which facilitates tumor cell invasion and metastasis (Chafe et al., 2021). While lactate creates an acidic environment conducive to tumor progression, it also promotes immune escape under these conditions. Bogdanov et al. (2022) confirmed that cancer cells establish a pH gradient in their microenvironment—acidic extracellularly and alkaline intracellularly—by activating and expressing proton and lactate transporters and exchangers. This low pH condition negatively impacts the functional activation of effector T cells, Tregs, and promotes their expression of programmed cell death receptor 1 (PD-1), thereby suppressing immune function. The low pH in the TME can downregulate the expression of inducible nitric oxide synthase (iNOS), chemokine ligand 2 (CCL2), and interleukin-6 (IL-6) in M1 macrophages while increasing the expression of M2 macrophage markers, thus inducing immune escape. The low pH in the TME can independently alter the phenotype and function of macrophages, particularly promoting M2 polarization (El-Kenawi et al., 2019; Pan et al., 2020). Pötzl et al. (2017) conducted an experiment using bicarbonate administration in a lymphoma mouse model. When the TME pH was increased from acidic (6.5–6.9) to physiological levels (7.2–7.5), NK cell production of IFN-γ increased, and tumor growth was delayed, demonstrating that the impact of the acidic TME on NK cell anti-tumor activity is reversible. Therefore, the acidic environment of the TME caused by lactate accumulation is not only a manifestation of acidosis but also a triggering factor for immune escape and tumor progression. Neutralizing this acidic environment offers a simple and feasible approach to restore immune cell activity.

#### 3.2.2 Lactate stimulates tumor angiogenesis

In the TME, the expression of vascular endothelial growth factor (VEGF) is directly influenced by the acidic microenvironment (Zhang et al., 2024b). High lactate levels can activate endothelial cells to produce VEGF and fibroblast growth factor-β (FGF-β) (Constant et al., 2000; Song et al., 2018). VEGF and FGF-β stimulate endothelial cell proliferation, migration, and angiogenesis, thereby promoting tumor cell proliferation, invasion, and drug resistance. Excessive lactate production can also induce TAMs to produce VEGFA, which further activates the formation of tumor lymphatic vessels and blood vessels (Milovanova et al., 2008).

In addition, lactic acid can interact with GPR81 and participate in tumor immune escape and angiogenesis as a signaling molecule. Tumor cells release lactic acid into the extracellular space via MCT4. Subsequently, lactic acid enters vascular endothelial cells through MCT1 and is converted into pyruvate, which activates the HIF-1α and nuclear factor-κB (NF-κB)/interleukin-8 (IL-8) signaling pathways, inducing endothelial cell migration and tumor angiogenesis (Doherty and Cleveland, 2013). After being taken up by MCT1 and oxidized to pyruvate, lactic acid can also regulate the expression and activity of inhibitor of κB kinase β (IKKβ), leading to the phosphorylation and proteasomal degradation of inhibitor of κBα (IκBα), nuclear translocation of NF-κB, and transcription of the angiogenic factor IL-8, ultimately promoting tumor angiogenesis (Duan et al., 2022). Studies have shown that the nanomaterial NanoMnSor, composed of a manganese dioxide (MnO_2_) core and a lipid-poly (lactic-co-glycolic acid) (PLGA) shell loaded with Sorafenib, can not only promote macrophage polarization to the M1 phenotype but also reduce VEGF concentration by generating a large amount of oxygen, thereby decreasing tumor vascularization (Ding et al., 2021). Annan et al. (2019) found that activation of the VEGF and its receptor signaling pathway increases the expression of carbonic anhydrase II (CA II) in tumor vascular endothelial cells, thereby affecting their proliferation and angiogenesis.

#### 3.2.3 The influence of lactic acid on cancer-associated fibroblasts

The activated fibroblasts in the TME, known as CAFs, reshape the extracellular matrix (ECM) to alter the TME structure, making it difficult for immune cells to infiltrate and enabling immune escape (Niu et al., 2024). Lactic acid can promote CAF generation by altering ECM composition and cell-cell interactions. Andreucci et al. (2023) found that lactic acid can induce the transformation of adipocytes and normal fibroblasts into myofibroblasts, causing them to lose adipocyte-specific markers such as fatty acid binding protein 4 (FABP4), CCAAT enhancer binding protein alpha (CEBPA), and peroxisome proliferator-activated receptor gamma (PPARγ). Simultaneously, these cells acquire myofibroblast markers including actin alpha 2, smooth muscle (ACTA2), collagen type I alpha 1 chain (COL1A1), and collagen type I alpha 2 chain (COL1A2), and increase mRNA expression levels of pro-inflammatory cytokines interleukin-1β (IL-1β) and IL-6, as well as the profibrotic factor transforming growth factor-β1 (TGF-β1). Fiaschi et al. (2012) demonstrated that in an acidic environment, CAFs increase glucose uptake, produce more lactic acid, and enhance lactic acid efflux through the expressed transporter MCT4. This lactic acid is then taken up by prostate cancer cells via MCT1 and used for the tricarboxylic acid (TCA) cycle and protein synthesis, promoting tumor cell proliferation and growth. The lactic acid-induced acidic environment plays a crucial role in remodeling both cellular and non-cellular components in the TME.

## 4 The role and mechanism of lactylation modification in tumors and the tumor microenvironment

### 4.1 The role and mechanism of lactylation modification and related proteins in tumors

Studies have shown that lactylation modification, including both histone and non-histone proteins, plays a significant cancer-promoting role in various tumors (Su et al., 2023; Zhang et al., 2023). In digestive system tumors, such as those of the stomach, intestine, and liver, Yang et al. (2022a) the lactylation level in gastric cancer tissues is significantly higher than in adjacent tissues and is associated with poor prognosis. Another study constructed a prognostic model based on lactylation modification levels in gastric cancer tissues, revealing that the lactylation score is closely related to overall survival and disease progression (Yang H. et al., 2023). Sun et al. (2023) demonstrated that the lactylation modification of methyltransferase METTL16 plays a crucial role in inducing copper death in gastric cancer cells. Miao et al. (2023) found that hypoxia-induced glycolysis enhances β-catenin stability and expression by promoting its lactylation, thereby exacerbating the malignant proliferation of colorectal cancer cells. Zhou et al. (2023) reported that GPR37 can enhance LDHA expression and glycolysis through the Hippo pathway, further increasing histone lactylation levels and promoting liver metastasis of colorectal cancer cells. Li et al. (2024b) recently showed that lactate promotes the expression of autophagy enhancer protein RUBCNL via histone lactylation, mediating resistance to bevacizumab in colorectal cancer cells. Wang J. et al. (2022) found that lipopolysaccharide (LPS) derived from intestinal bacteria can increase the level of long non-coding RNA - LINC00152 by inducing histone lactylation, further promoting the invasion and migration of colorectal cancer. In addition, Wu (2023) identified specific lactylation sites on USP14 and ABCF1 proteins as diagnostic indicators for liver cancer and its metastasis after omics analysis of lactylation modifications in liver cancer. Yang et al. (2023b) found that lactylation modification preferentially affects enzymes involved in metabolic pathways after analyzing lactylation modification in hepatitis B virus-related liver cancer samples, and further confirmed that lactylation modification can promote the proliferation and metastasis of liver cancer cells by inhibiting the function of AK2. Cheng et al. (2023) demonstrated that lactylation-related genes can serve as biomarkers for the clinical treatment effect of liver cancer. In urinary system tumors, such as kidney and bladder cancers, Yang J. et al. (2022) found that histone lactylation levels are associated with poor prognosis in clear cell renal cell carcinoma (ccRCC) patients. Mechanistic studies revealed that histone lactylation promotes ccRCC progression by activating platelet-derived growth factor receptor β (PDGFRβ) transcription. Targeting histone lactylation can inhibit ccRCC cell proliferation and metastasis *in vivo*. Xie et al. (2023) found that the circXRN2-Hippo pathway regulatory axis regulates tumor progression in bladder cancer by inhibiting histone lactylation and LCN2 protein expression. In addition, Wang X. et al. (2023) reported that histone lactylation promotes the proliferation of undifferentiated thyroid cancer with BRAF mutation, and the combined use of lactate metabolism inhibitors and BRAF V600E inhibitors effectively inhibits thyroid cancer cell proliferation. He et al. (2023) found that loss of the Numb/Parkin pathway in prostate cancer and lung adenocarcinoma leads to metabolic reprogramming, causing increased intracellular lactate levels, which in turn increase histone lactylation and activate gene transcription related to neuroendocrine function. In ocular melanoma, Yu et al. (2021) found that histone lactylation promotes the degradation of PER1 and TP53 mRNA by enhancing the transcription of the RNA N6-methyladenosine (m6A) modification recognition protein YTHDF2, ultimately improving the proliferation and migration ability of ocular melanoma cells.

It should be noted that lactylation modification may also exert a tumor-suppressing role. Jiang et al. (2021) found in their study of non-small cell lung cancer (NSCLC) that an increase in histone lactylation levels can lead to decreased expression of hemokinin-1 (HK-1) and pyruvate kinase M (PKM) involved in glycolysis, while increasing the levels of succinate dehydrogenase (SDHA) and isocitrate dehydrogenase 3γ (IDH3γ) in the TCA cycle. This results in the inhibition of glucose uptake and glycolysis in tumor cells, reducing cell proliferation and migration. Longhitano et al. (2022) found that lactate-induced histone H3K18la modification can lead to increased homozygosity, enlarged nuclei, and cell cycle arrest in uveal melanoma (UM) cells, thereby inhibiting UM progression. Additionally, recent research has made progress in understanding the role of lactylation modification-related enzymes in tumors. Zu et al. (2022) found that histone delactylase SIRT2 can inhibit the proliferation and migration of neuroblastoma cells. Jin et al. (2023) reported that delactylase SIRT3 can inhibit the proliferation of liver cancer cells by regulating the lactylation modification level of Cyclin E2. The above studies have revealed the role and mechanism of lactylation modification and related proteins in cancer progression and treatment, and can provide new markers and targets for the clinical diagnosis and targeted therapy of tumors. It is important to note that the specific functions of lactylation modification and related proteins may vary depending on the type of cancer, necessitating targeted research to explore their precise roles and underlying molecular mechanisms.

### 4.2 The role and mechanism of lactylation modification in the tumor microenvironment

**FIGURE 2:**
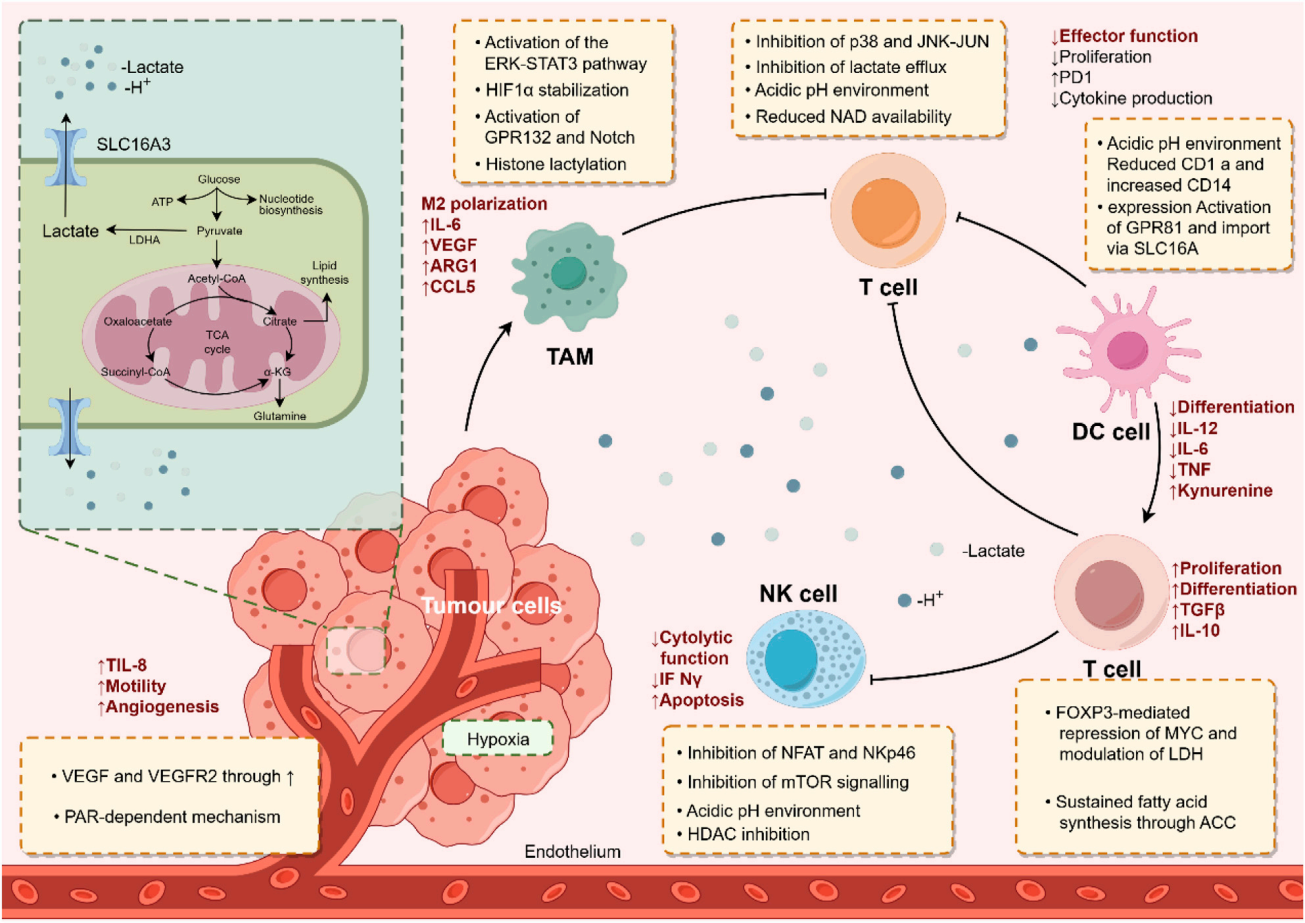
The immunoregulatory role of lactate and lactylation modification in the tumor microenvironment. By Figdraw.

The TME is crucial for the occurrence and development of tumors. Existing studies have shown that, in addition to tumor cells, lactate and lactylation modification also play significant roles in other cellular components within the TME (Qu et al., 2023). Immune cells are a major category of cellular components in the TME and play an important role in tumor cell immune escape and immunotherapy. Lactate in the TME can regulate the metabolism of immune cells, inhibiting the proliferation and function of CD8^+^ T cells, NK cells, dendritic cells, etc., thereby mediating immune escape (Brown and Ganapathy, 2020; Tu et al., 2021). Macrophages, as key innate immune cells in the TME, are divided into two phenotypes based on their functions: M1, which is pro-inflammatory and anti-cancer, and M2, which is anti-inflammatory and pro-cancer. Zhang et al. (2019) found that lactate can induce an increase in M2 phenotype-related proteins such as VEGF through histone lactylation modification, promoting the transformation of macrophages from the M1 to the M2 phenotype. Wang L. et al. (2022) discovered that the key molecule proprotein convertase subtilisin/kexin type 9 (PCSK9), highly expressed in colon cancer, regulates lactate levels and macrophage migration inhibitory factor (MIF) expression. Knockdown of PCSK9 reduces lactate secretion by tumor cells and MIF expression, further promoting M1 polarization of TAMs and inhibiting their M2 polarization. Chaudagar et al. (2023) f found in prostate cancer that decreased phosphoinositide 3-kinase (PI3K) levels lead to reduced lactate synthesis in tumor cells, inhibiting histone lactylation modification in TAMs and enhancing their immune efficacy. Tumor-infiltrating myeloid cells (TIMs), a type of innate immune cell closely related to tumor immune escape, are key regulators of tumor progression. Xiong et al. (2022) found in rectal cancer that lactate in the TME promotes the expression of RNA N6-methyladenosine (m6A) methyltransferase METTL3 in TIMs via histone lactylation modification, further playing a cancer-promoting role by enhancing RNA m6A modification and activating the JAK1-STAT3 signaling pathway. In addition, regulatory T cells (Treg) in the TME play a crucial role in maintaining an immunosuppressive microenvironment (also known as a “cold” TME). Gu et al. (2022) found that lactylation modification of the membrane organizing protein Moesin mediates Treg generation by increasing its interaction with the transforming growth factor-β (TGF-β) receptor and activating the SMAD3 signaling pathway, further promoting tumor immune escape. Inhibiting Moesin lactylation can improve the efficacy of immunotherapy. Wang Z.-H. et al. (2023) identified a special FOXP3^+^ NKT-like cell in the “cold” TME of malignant pleural effusion. Single-cell sequencing analysis revealed that these FOXP3^+^ NKT cells highly express MCT and lactate dehydrogenase B (LDHB) to uptake and utilize lactate, thereby maintaining their immunosuppressive function. These findings reveal that lactate and lactylation modification play an important role in the regulation of immune cell functions in the TME and can provide new ideas for overcoming immunosuppression and improving the effect of tumor immunotherapy (Figure 2). In addition, the role and mechanism of lactylation modification in other cellular components with important functions in the TME such as CAFs and endothelial cells have not been reported yet and need to be further explored.

## 5 Therapy targeting lactylation modification

Research on lactylation modification and its related enzymes indicates that targeting lactylation modification represents a novel strategy for inhibiting tumor progression and enhancing anti-tumor effects, providing a new target for the development of anti-tumor drugs (Fan et al., 2023). Targeting lactylation modification can focus on key proteins involved in processes such as lactate metabolism, transport, or lactylation modification itself. Current research primarily centers on inhibitors of proteins related to lactate metabolism and transport.

### 5.1 Inhibitors related to lactate metabolism

Inhibiting the activity of key enzymes in intracellular lactate metabolism can effectively reduce lactylation modification by lowering lactate levels. LDHA is a critical enzyme that converts pyruvate to lactate during glycolysis, and its upregulation is associated with poor prognosis in various malignant tumors. Several effective LDH inhibitors have been identified. For example, Oxamate acts as a pyruvate-competitive inhibitor of LDHA and has been shown to inhibit tumor cell proliferation (Zhao et al., 2015). NADH-competitive inhibitors of LDHA, such as Gossypol (also known as AT-101), FX11 (3-dihydroxy-6-methyl-7-(phenylmethyl)-4-propylnaphthalene-1-carboxylic acid), and quinoline-3-sulfonamide, have also been proven to inhibit tumor cell proliferation (Van Poznak et al., 2001; Billiard et al., 2013). Galloflavin inhibits LDHA activity through direct binding, reducing lactate production and inducing apoptosis in tumor cells (Manerba et al., 2012). However, due to the non-specific effects of LDHA inhibitors and their complex interactions with other cellular components, indiscriminate inhibition of LDHA activity may lead to uncontrollable side effects, thereby limiting their clinical development. Dichloroacetate (DCA) is an oral small molecule drug that inhibits pyruvate dehydrogenase kinase (PDK), promoting glucose oxidation in glycolysis and activating mitochondrial apoptosis. This results in the inhibition of tumor cell proliferation and has shown efficacy in glioblastoma and advanced head and neck squamous cell carcinoma (Michelakis et al., 2008; Dunbar et al., 2014; Powell et al., 2022). Beyond affecting intracellular lactylation modification levels, lactate metabolism-related inhibitors have broader and more complex impacts on intracellular metabolic reactions. Therefore, improving the specificity of these inhibitors, minimizing side effects, and analyzing their effects on specific lactylation modifications are critical challenges for future development in targeted lactylation modification therapy.

### 5.2 Lactate transporter inhibitors

MCTs play a crucial role in maintaining metabolic homeostasis within the TME. They ensure the maintenance of glycolysis and an acid-resistant phenotype, thereby promoting the malignant behavior of cancer cells (Fakhri et al., 2022). MCTs facilitate rapid glycolysis by enabling the export of lactic acid and are involved in pH regulation through the co-transport of protons (Li et al., 2022). MCT1 and MCT4 are the most widely expressed MCT subtypes in cancer cells (Payen et al., 2020). MCT1 has a high affinity for lactic acid and is preferentially expressed in oxidative cancer cells that absorb lactic acid (Zhang et al., 2024a). In contrast, MCT4 has a lower affinity for lactic acid but a higher turnover rate, making it particularly suitable for promoting lactic acid export from glycolytic cancer cells. Its expression is upregulated under hypoxic conditions, regulated by HIF-1α (Yamaguchi et al., 2023). Moreover, MCTs also promote the migration and invasion of tumor cells. Izumi et al. (2011) demonstrated that the expression of MCT1 and MCT4 is associated with the invasion of human lung cancer cells, indicating their potential as target proteins for cancer therapy. Inhibiting the activities of MCT1 and MCT4 can achieve therapeutic goals (Payen et al., 2020). AR-C155858 is a dual inhibitor of MCT1 and MCT2 that inhibits T lymphocyte activation by blocking lactic acid excretion, thereby exerting immunosuppressive effects. AR-C155858 binds to transmembrane domains 7-10 (TM7-10) of MCT1 intracellularly to inhibit its activity (Ovens et al., 2010a). It effectively inhibits MCT1 in rat red blood cells expressing endogenous MCT1, with a Ki value of 2.3 nmol/L. In studies on *Xenopus laevis* oocytes transfected with MCT2, AR-C155858 has been shown to inhibit MCT2, albeit less potently (K_i_>10 nmol/L) (Ovens et al., 2010b). The inhibitory effect of AR-C155858 on MCT2 is selective; it inhibits MCT2 when bound to the auxiliary protein Basigin but not when bound to the preferred partner protein Embigin (Ovens et al., 2010b). AZD3965, a derivative of AR-C155858, is also a dual inhibitor of MCT1 and MCT2. It effectively inhibits lactic acid excretion in tumor cells, suppresses tumor growth, and enhances radiosensitivity. AZD3965 exhibits six times greater potency against MCT1 compared to MCT2 and has no inhibitory effect on MCT3 or MCT4 at concentrations up to 10 μmol/L (Bola et al., 2014). AZD3965 has been shown to significantly reduce cell and tumor growth in human small cell lung cancer and various lymphoma xenograft models overexpressing MCT1 (Izumi et al., 2011; Polański et al., 2014; Noble et al., 2017). Currently, a phase I clinical trial (NCT01791595) for advanced solid tumors and lymphomas is underway in the UK (Guan et al., 2019). In the future, it may be used in the treatment of prostate cancer, gastric cancer, and diffuse large B-cell lymphoma.

The novel inhibitor BAY-8002 effectively inhibits the bidirectional transport of lactic acid, significantly increasing intratumoral lactic acid levels and transiently regulating pyruvate levels, thereby placing tumor cells in a stagnant state. BAY-8002 is five times more selective for MCT1 than for MCT2 and has no inhibitory effect on MCT4. Radiolabeling studies investigating the competitive inhibition of MCT1 by AZD3965 and BAY-8002 revealed that these two ligands share similar binding sites, indicating they have comparable modes of action (Quanz et al., 2018). 7-Aminocarboxycoumarin (7ACC) has been developed to selectively interfere with lactic acid flux in the lactic acid-rich tumor microenvironment. Specifically, 7ACC2 inhibits lactic acid influx in cancer cells expressing MCT1 and MCT4 but does not affect efflux, suggesting its mode of action differs from that of AR-C155858 (Draoui et al., 2014). This indicates that 7ACC selectively targets one part of the MCT transporter translocation cycle, leading to strict inhibition of lactic acid influx. This unique activity is associated with anti-tumor effects and is less prone to drug resistance and side effects. Additionally, the potent MCT4 inhibitor VB124 has enhanced anti-PD-1 treatment efficacy in hepatocellular carcinoma (HCC) xenografts. Studies show that MCT4 expression is higher in immune checkpoint therapy (ICT)-resistant patients compared to responders (Fang et al., 2023).

**FIGURE 3:**
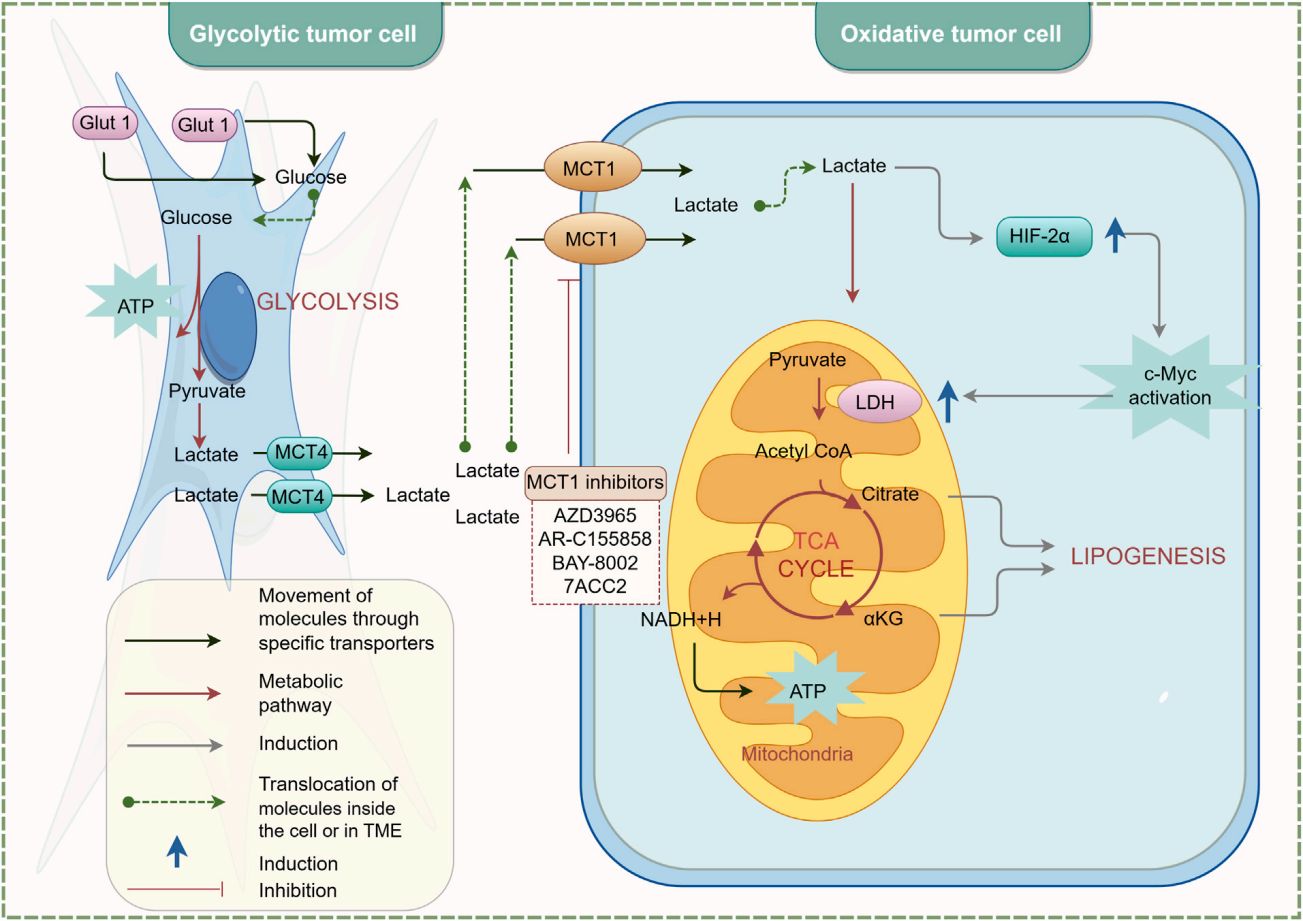
Inhibition mechanism of MCT. By Figdraw.

Wang N. et al. (2021) resolved the cryo-electron microscopy structures of the wild-type (WT) human MCT1/Basigin-2 complex and three prototype MCT inhibitors, AZD3965, BAY-8002, and 7ACC2, revealing the precise binding sites and inhibitory mechanisms of these inhibitors. The structure of MCT1-Basigin-2 in complex with lactic acid exhibits an outward-open conformation. Among the inhibitor-bound complexes, MCT1-Basigin-2 bound to BAY-8002 and AZD3965 also adopt an outward-open conformation, whereas the complex with 7ACC2 is in an inward-open state. Despite these differences, all three inhibitors directly occupy the substrate-binding site and inhibit MCT1 through direct competition with substrate binding and inhibition of transporter conformational changes. These findings elucidate the modes of action of three candidate anti-cancer drugs, providing a critical framework for structure-guided drug discovery targeting MCT. The inhibitory mechanism of MCT is illustrated in Figure 3. In the future, further determination of the specific roles played by MCTs in different cancers and the lactylation modification process, as well as their structural basis and regulatory mechanisms, will provide important evidence for the development of more specific and targeted MCT inhibitors for clinical use.

### 5.3 The role of targeting lactylation modification in tumor immunotherapy

In addition, lactylation modification plays a crucial role in tumor immunotherapy. The efficacy of PD-1 blockade immunotherapy is determined by the competition between the activation of CD8^+^ T cells and PD-1-expressing Treg cells in the TME. Gu et al. (2022) found that lactate promotes tumor progression by regulating the lactylation modification of MOESIN in Treg cells, and patients with hepatocellular carcinoma who respond effectively to PD-1 antibody treatment exhibit low lactylation levels in their Treg cells. Kumagai et al. (2022) discovered that in malignant tumors with high glycolysis levels, Treg cells rapidly absorb lactate from the TME via MCT1, stimulating activated nuclear factor of activated T cells 1 (NFAT1) to enter the nucleus and increase PD-1 expression, while inhibiting PD-1 expression in effector T cells, ultimately leading to treatment failure. Lactate can also promote the expression of programmed cell death ligand 1 (PD-L1) on macrophages and neutrophils, inducing immune resistance (Deng et al., 2021). Weng et al. (2019) reported that targeting the MCT1/miR-34a/IL-6/IL-6R signaling axis can inhibit M2 polarization of macrophages in triple-negative breast cancer. Preclinical studies have shown that combining the MCT1 inhibitor AZD3965 with anti-PD-1 therapy reduces lactate release into the TME and enhances anti-tumor immunity. Additionally, the combination of anti-PD-1 drugs and LDHA inhibitors exhibits a stronger anti-tumor effect compared to anti-PD-1 drugs alone (Huang et al., 2021). These findings suggest that combining inhibitors of lactylation modification with immunotherapy may provide a new strategy for combined tumor treatment.

All the above studies indicate that targeting lactate metabolism and lactylation modification has emerged as a promising and forward-looking treatment strategy. Therefore, exploring lactylation modification and its regulatory sites can further identify effective cancer treatment targets and provide new directions for combination therapy. However, although increasing evidence suggests that lactate is a viable target for anti-tumor and sensitizing treatments, the role and mechanisms of lactylation modification in these processes still require further elucidation. Additionally, current interventions targeting lactylation modification primarily focus on inhibiting lactate production, transport, and signal transduction, but their specificity and effectiveness for lactylation modification need improvement. Continuing to explore and identify specific “writer,” “eraser,” or “reader” proteins involved in lactylation modification is a key task for truly targeting this process and providing new therapeutic targets for cancer treatment.

## 6 Conclusion

Histone lactylation modification is a novel post-translational modification of proteins that plays a crucial role in the occurrence and development of cancer. It regulates tumor progression by influencing cancer cell proliferation, invasion, migration, metabolic reprogramming, epigenetic regulation, signal pathway modulation, and microenvironmental interactions. In-depth research into the mechanisms of histone lactylation reveals that this modification is an extremely dynamic biological process with significant regulatory effects on chromatin structure and gene transcription. Moreover, lactylation modification interacts with other epigenetic modifications (such as methylation, phosphorylation, etc.) to jointly regulate gene expression and cellular physiological functions.

Lactylation modification can influence cancer progression and treatment outcomes by regulating the physiological functions of tumor cells, cancer stem cells, and immune cells within the TME. It has emerged as a new target for cancer therapy, but several limitations and challenges remain: 1) Lack of a standardized method for detecting lactylation modification. Currently, various methods are used to detect lactylation modification, including Western blotting, immunohistochemistry, and mass spectrometry. Each method has its own advantages and limitations. The absence of a standardized detection protocol leads to inconsistent results and hinders comparative studies. 2) The complexity of lactylation modification metabolism and signal pathways. Lactylation modification is involved in multiple metabolic processes and signaling pathways. Although several signaling pathways related to lactylation have been identified, its precise role in these pathways remains incompletely understood. Identifying specific biomarkers or targets for inhibiting lactylation modification remains a significant challenge. Further research is needed to elucidate the exact molecular mechanisms of lactylation modification in colorectal cancer (CRC) and other cancers, facilitating the translation of lactylation modification research from basic science to clinical application.

Currently, the regulatory mechanisms of lactylation modification are still in the preliminary research stage. The cause of histone lactylation may be attributed to lactate accumulation or substances similar to lactate, but this remains an open question requiring further investigation. Lactate is continuously produced in various cell types throughout the body. However, it remains unclear whether histone lactylation can be structurally utilized in different cells, tissues, and organs except under pathological conditions. Although many challenges remain, emerging evidence linking lactylation modification to cancer suggests that histone lactylation as a biomarker holds broad prospects for cancer research and treatment. Investigating the function and regulatory mechanisms of lactylation modification in physiological and pathological processes is crucial for deepening our understanding of disease onset and progression, including tumors. Lactylation modification bridges metabolism with gene expression and is expected to become a breakthrough in treating tumors at the genetic level. Overall, histone lactylation modification not only provides a new perspective and strategy for studying PTMs but also opens up new directions for research in oncology and immunology. These findings facilitate the development of this field, help identify novel therapeutic approaches targeting histone lactylation, and may promote the research and development of new anti-cancer drugs.
